# Glia-Derived Extracellular Vesicles: Role in Central Nervous System Communication in Health and Disease

**DOI:** 10.3389/fcell.2020.623771

**Published:** 2021-01-25

**Authors:** Cristiana Pistono, Nea Bister, Iveta Stanová, Tarja Malm

**Affiliations:** A.I. Virtanen Institute for Molecular Sciences, University of Eastern Finland, Kuopio, Finland

**Keywords:** extracellular vesicle, glia, central nervous system, astrocyte, oligodendrocytes, microglia

## Abstract

Glial cells are crucial for the maintenance of correct neuronal functionality in a physiological state and intervene to restore the equilibrium when environmental or pathological conditions challenge central nervous system homeostasis. The communication between glial cells and neurons is essential and extracellular vesicles (EVs) take part in this function by transporting a plethora of molecules with the capacity to influence the function of the recipient cells. EVs, including exosomes and microvesicles, are a heterogeneous group of biogenetically distinct double membrane-enclosed vesicles. Once released from the cell, these two types of vesicles are difficult to discern, thus we will call them with the general term of EVs. This review is focused on the EVs secreted by astrocytes, oligodendrocytes and microglia, aiming to shed light on their influence on neurons and on the overall homeostasis of the central nervous system functions. We collect evidence on neuroprotective and homeostatic effects of glial EVs, including neuronal plasticity. On the other hand, current knowledge of the detrimental effects of the EVs in pathological conditions is addressed. Finally, we propose directions for future studies and we evaluate the potential of EVs as a therapeutic treatment for neurological disorders.

## Introduction

The human central nervous system (CNS) is an extremely complex organ composed by billions of highly specialized cells. In this review, we focus on two main types of cells: neurons, electrically active cells, which form a complex circuitry for the transmission and the processing of information, and glial cells. Glia are spread throughout the CNS and are essential for its homeostasis. Glia comprise a heterogeneous population of cells with different structures, functions and origin, and play a key role in pivotal physiological conditions, by performing housekeeping functions (Allen and Lyons, 2018). Moreover, when environmental stress or pathological conditions challenge CNS homeostasis, glial cells respond, with the attempt to defend CNS and preserve its normal functions. Glia are divided into macroglia and microglia. Macroglia consist of astrocytes and oligodendrocytes, cells of neuroepithelial origin (Rowitch and Kriegstein, 2010). Astrocytes are a heterogeneous population of cells with critical functions: they support neurons and are essential for the correct synaptic activity and modulation of synaptic plasticity (Mederos et al., 2018). Moreover, astrocytic processes create contacts not only with neurons, but also with blood vessels, aiding in the maintenance of blood-brain barrier (BBB) integrity (Abbott et al., 2010; Farhy-Tselnicker and Allen, 2018). Oligodendrocytes are responsible for production of myelin, thus maximizing the conduction of neuronal impulse through the axons (Simons and Nave, 2016). Microglia are cells of myeloid origin (Tay et al., 2017). Although they account for only about 10% of glia and are small in size, they are big in their function: they monitor and protect the CNS environment for the presence of tissue infection and damage (Wolf et al., 2017), control neuronal connectivity, synaptic transmission and plasticity, and prune synapses during development (Tay et al., 2017).

All the roles carried out by neurons and glia are tightly controlled and organized by an intricate communication system which can be mediated by direct contact-dependent mechanisms and paracrine action of secreted molecules (Eyo and Wu, 2013; Araque et al., 2014). During the last decades, the importance of extracellular vesicles (EVs) for this cell-to-cell communication has become evident: these membrane structures allow the transfer of molecules to other cells locally or over longer distances. Indeed, EVs can deliver active molecules across the BBB, making them a potential tool for early diagnosis of neurological diseases (Fiandaca et al., 2015) and potential vehicles for targeted and non-invasive therapies (Alvarez-Erviti et al., 2011).

The research on the physiological role of EVs in the CNS and on their involvement in CNS disorders, together with the potential role of EVs in treating these diseases, is rapidly progressing. In this review we describe the role of glia-derived EVs in the inter-cellular communication between glia and neurons, considering both physiological and pathological mechanisms in which EVs may be involved in the CNS.

## Extracellular Vesicles in the Central Nervous System

EVs are double membrane-enclosed vesicles released into the extracellular space by potentially all cells, including neurons, astrocytes, oligodendrocytes, and microglia (Potolicchio et al., 2005; Fauré et al., 2006; Krämer-Albers et al., 2007; Proia et al., 2008), and can be found in practically all body fluids (Pisitkun et al., 2004; Ogawa et al., 2016; Karimi et al., 2018; Manek et al., 2018). EVs are often divided into two main subtypes: exosomes that originate from the endosomal pathway after the fusion of the multivesicular bodies with the plasma membrane, and microvesicles that are shed through outward budding of the plasma membrane. Exosomes are characterized by a size between 30 and 150 nm, whereas microvesicles have a size between 100 and 1000 nm (Vidal, 2019). Exosomes and microvesicles are highly heterogeneous groups, both in terms of molecular and biological properties (Willms et al., 2016; Van Niel et al., 2018). This, together with the overlap in size, makes it difficult to certainly assign the biogenetic origin after their release from the cell (Hartjes et al., 2019). Moreover, no concrete standardization for EV isolation and characterization, that would fit in all circumstances, exist, which adds an additional challenge in the interpretation of the conclusions derived from EV studies. In recent years, the International Society of Extracellular Vesicles has started extensive work to compile common guidelines for the field to avoid artifacts or misinterpretation when analyzing the functions of EVs, and we highly recommend the reader to refer to these guidelines (Théry et al., 2018). The methods used to isolate EVs include ultracentrifugation, ultrafiltration, size exclusion chromatography, polymer precipitation, immunoaffinity capture, and microfluidic techniques, to name a few. All methodologies have both advantages and disadvantages (Yang et al., 2020) and the chosen method can vary depending on the type of biological sample, the purpose of the analysis and the cost (Diaz et al., 2018; Patel et al., 2019; Guzman and Guzman, 2020). Being extensively reviewed elsewhere, we kindly suggest the reader to refer to the aforementioned literature on the importance of EV isolation methodology selection.

The content of both exosomes and microvesicles varies based on the type of cell from where they derive, and can change based on the physiological state of the releasing cell and on environmental *stimuli* (Yáñez-Mó et al., 2015). Initially, EVs were considered as carriers of waste products, but now it is clear that their role goes beyond this: EVs are responsible for the exchange of molecules, including proteins, lipids, and nucleic acids, allowing the transfer of signals and contributing to the maintenance of cellular homeostasis (Vidal, 2019). EVs can reach the target cells by fusion with the plasma membrane, or by internalization through endocytosis, macropinocytosis or phagocytosis. In this way, EVs have been described to be involved in the mediation of a plethora of processes, including immune response, stem cell activation, and response to stress (Anel et al., 2019; Bruno et al., 2019; O'Neill et al., 2019). EVs have also a role during CNS development and later they continue to contribute to its homeostasis, being, for example, involved in waste elimination, trophic support of neurons, antigen presentation and maintenance of myelin and synaptic plasticity (Krämer-Albers et al., 2007; Fitzner et al., 2011). The involvement of EVs in the CNS homeostasis appears to be central for also non-mammalians. Indeed, considerable evidence for the functional role of EVs has been collected from invertebrate models, which exhibit developmental and physiological mechanisms in the nervous systems that are highly conserved across various animals. For example, glia-derived EVs transport the miR-274, a microRNA required for the coupling of synaptic boutons to tracheal branches in Drosophila larvae (Tsai et al., 2019). In addition, the medicinal leech CNS constitute an interesting model to study the interaction between microglia and neurons, and microglia-derived EVs play a role in this process, exhibiting neurotrophic properties (Raffo-Romero et al., 2018). Interestingly, leech microglia EVs have been described to trigger a significant increase of rat PC12 cell differentiation, suggesting the presence of common molecular mediators and the evolutionary conservation of EV-mediated communication systems (Raffo-Romero et al., 2018). In addition, a proteomic analysis and studies on miRNA signatures of EVs released from leech microglia further support the presence of conserved neuroprotective cargo throughout the evolution (Arab et al., 2019; Lemaire et al., 2019).

It is essential to take into account that the role of EVs in the CNS can goes beyond the maintenance of homeostatic functions, as they can be involved in the pathogenesis of CNS disorders (Nikitidou et al., 2017) and in the communication between the cells of the microenvironment of CNS tumors such as glioblastoma (Simon et al., 2020). However, understanding the impact of EVs on the complexity of CNS disorders is not easy, as suggested by the fact that EVs can contribute to the removal of toxic proteins and aggregates, but, on the other hand, they can be involved in the spread of pathogenic proteins (Ngolab et al., 2017; Sardar Sinha et al., 2018). Thus, better knowledge of the source of EVs and their impact on the specific CNS cell types, including neurons, is essential, not only to understand the involvement of EVs in physiological processes of the CNS, but to deepen our knowledge on their pathological role in neurodegenerative and neuroinflammatory diseases. To this regard, next we will focus on the role of EVs from different glial cells in several central mechanisms of CNS function, and on the implication of EVs for the pathogenesis of CNS disorders.

## Role of Astrocyte-Derived EVs in the Central Nervous System

Astrocytes are the most abundant cell type in the CNS and play a key role in its homeostasis, by regulating BBB permeability, nutrient uptake, and removal of waste metabolites. In addition, they protect neurons from cell death and neurotoxicity, and play a role in regulating neurogenesis and synaptogenesis (Allen and Lyons, 2018; Michinaga and Koyama, 2019). These cells have a regulatory role also at the astrocyte-microglia level by modulating microglial phenotypes and phagocytosis (Vainchtein and Molofsky, 2020). Under physiological conditions, astrocytes respond to the neuronal activity, by sensing several neurotransmitters. In turn, they can release a variety of biomolecules, which can selectively target neurons, thus contributing to their maturation and survival, and to the modulation of synaptic function (Durkee and Araque, 2019). Astrocytes also react to pro-inflammatory molecules released from other CNS cells and are able to mediate inflammatory responses (Giovannoni and Quintana, 2020), highlighting the diversity of processes in which astrocytes are involved.

### Effects of Astrocyte-Derived EVs on Neurons

**Figure 1:**
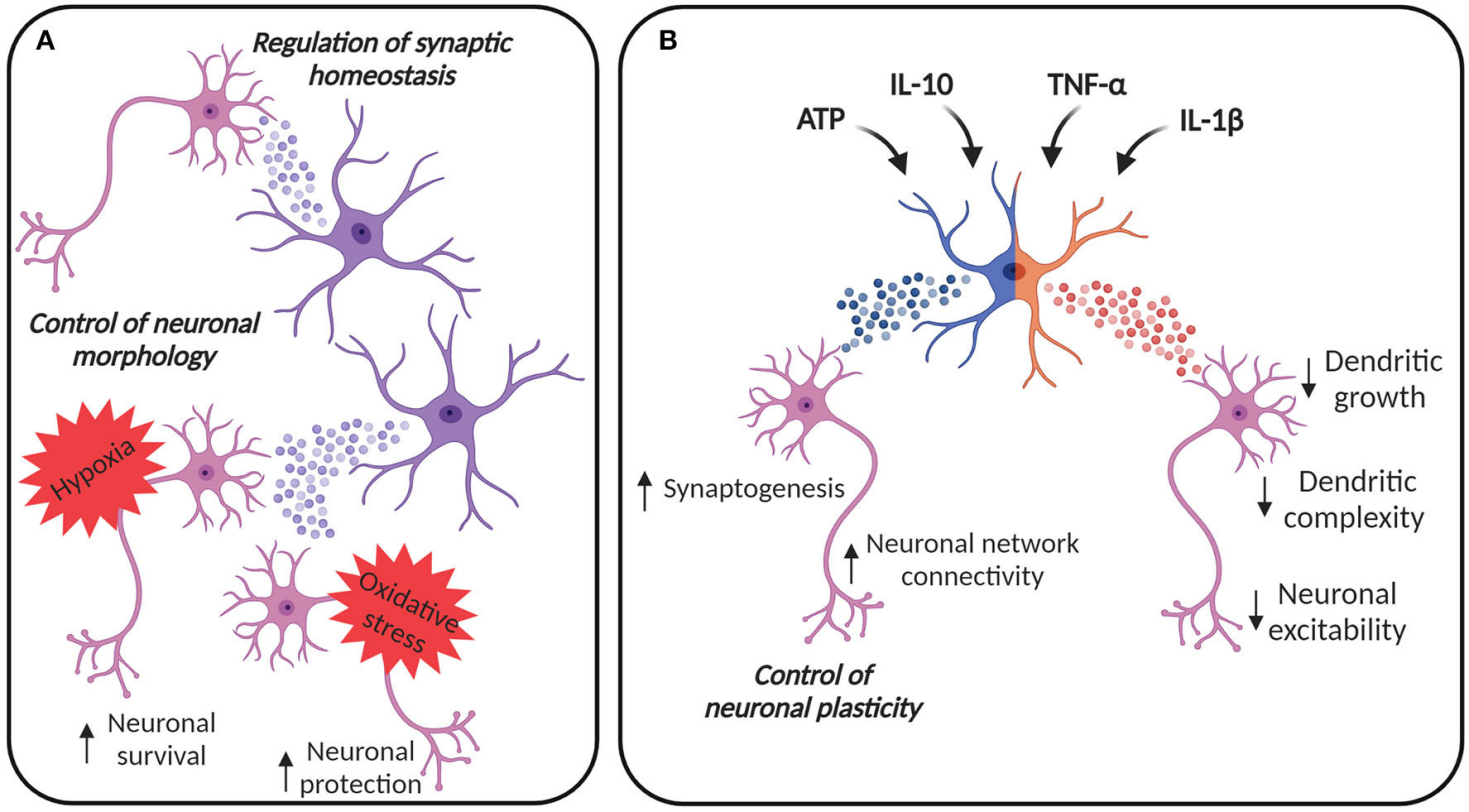
EVs in astroglia-neuron communication. **(A)** EVs from astrocytes can be internalized by neurons, contributing to the control of cellular morphology and synaptic homeostasis. Astrocyte-derived EVs can contribute to neuronal survival under hypoxic and ischemic conditions and to the protection against oxidative stress. **(B)** Astrocytes can adapt their EV content in response to different *stimuli*. EVs from astrocytes stimulated with ATP and anti-inflammatory *stimuli*, like IL-10, may promote synaptogenesis and may increase neural network connectivity, thus contributing to the regulation of neuronal plasticity. On the other hand, astrocytes are also immune-competent cells, able to respond to inflammatory molecules. IL-1β and TNF-α increase the release of EVs from astrocytes, vesicles that are enriched with miRNAs and proteins able to reduce dendritic growth in developing neurons, simplify dendritic complexity in mature neurons, finally damping neuronal excitability. Created with BioRender.com.

Communication between astrocytes and neurons is possible due to the release of several molecules, including neurotransmitters, and EVs additionally contribute to this process. For example, EVs derived from astrocytes can transport mtDNA (Guescini et al., 2010) and specific miRNAs, with a profile that differ from the one of astrocytes (Jovičić and Gitler, 2017). Importantly, the alteration of the miRNAs may have an impact on CNS development and function, highlighting the centrality of EVs for the correct cell-to-cell communication (Jovičić and Gitler, 2017). Several studies have suggested that astrocyte-derived EVs are neuroprotective. For example, EVs released from astrocytes contain the angiogenic factors VEGF and FGF-2, suggesting their contribution to brain cell differentiation and function (Proia et al., 2008). EVs can be released from astrocyte processes, acutely prepared from adult rat cerebral cortex, possibly contributing to the signal transmission in the CNS. Indeed, these EVs can selectively target neurons in co-culture and they can be internalized (Venturini et al., 2019). Astrocyte-derived EVs have been shown to exhibit neuroprotection by prion protein (PrP) dependent mechanisms against hypoxia, ischemia and hypoglycemia (Guitart et al., 2016). EVs can carry PrP from astrocytes to PrP deficient neurons and thereby enhance neuronal survival (Guitart et al., 2016). Moreover, ApoD-containing EVs secreted by astrocytes may mediate protection of neurons against oxidative stress, a challenge typical of aging and several pathological conditions (Pascua-Maestro et al., 2018) (Figure 1A).

EVs from astrocytes exposed to oxygen and glucose deprivation (OGD) preconditioning can also reduce neuronal cell death (Xu et al., 2019) (Figure 1A). Xu et al. showed that miR-92b-3p levels, an oncogenic miRNA promoting cell proliferation (Zhuang et al., 2016), were increased in the EVs, suggesting this miRNA as a mediator for subsequent protection of neurons from OGD injury. In the context of ischemic stroke, Pei at al. described that EVs from astrocytes are able to inhibit the apoptosis in neurons subjected to OGD by regulation of autophagy (Pei et al., 2019). A follow-up study showed the involvement of miR-190b in this protection: astrocytes secrete miR-190b into the EVs and, when taken up by neurons, EV-containing miR-190b regulates autophagy (Pei et al., 2020). In addition, EVs shed from the astrocytes have been studied in the context of traumatic brain injury (TBI), in which they can contribute to protection and repair damaged neurons through restoration of mitochondrial function and downregulation of apoptosis (Chen et al., 2020).

#### Impact of Astrocyte-Derived EVs on Neuronal Morphology and Synaptic Plasticity

Astrocyte-derived EVs can directly impact neurons by regulating their morphology, as shown in hippocampal neurons (Luarte et al., 2020) (Figure 1A). The regulation of dendritic development may be dependent on astrocytes' ability to modify the miRNA cargo in their EVs (Luarte et al., 2020).

Chaudhuri et al. described that astrocytes can release EVs enriched with miRNAs that regulate synaptic homeostasis (Chaudhuri et al., 2018) (Figure 1A). Some of these miRNAs, such as miR-29c and miR-130a, were previously described to target mRNAs involved in the regulation of neurite outgrowth, dendritic spine formation, and density (Zou et al., 2015; Zhang et al., 2016), suggesting that the constitutive release of EVs from astrocytes may participate in the homeostatic maintenance of neuronal synapses. Furthermore, astrocytes treated with ATP may promote neuronal survival, synapse maturation and may increase neural network connectivity, through induced release of EVs with specific miRNA cargo such as miR-21 and miR-29a known to regulate axon growth and neuronal survival. Coherently, astrocytes can also alter their EV protein cargo in response to different *stimuli*. A recent study compared the proteomic profile of EVs from astrocytes treated with ATP, interleukin (IL)-1β and IL-10, showing that EVs from astrocytes stimulated with ATP or anti-inflammatory IL-10 are enriched with proteins involved in neurite outgrowth, axonal guidance, synaptogenesis, and synaptic long-term potentiation (Datta Chaudhuri et al., 2020). Moreover, EVs from astrocytes treated with IL-10 contain proteins that can regulate gap junction signaling and CREB signaling (Datta Chaudhuri et al., 2020). Considering that gap junction plasticity is a homeostatic mechanism to balance asynchronous irregular neuronal activity and synchronized oscillations (Pernelle et al., 2018), and CREB signaling is involved in the enhancement of long-term synaptic plasticity and neuronal excitability (Zhou et al., 2009) as well as in the increment of spine density and in the alteration of local neuronal connectivity (Sargin et al., 2013), it is clear that these EVs are likely to have a role in the regulation of neuronal plasticity (Figure 1B).

### Effects of Astrocyte-Derived EVs on Neurons During Neuroinflammation

Astrocytes act also as immune-competent cells, able to respond to and activate immune responses, by secreting cytokines and chemokines. TNF-α treatment promotes the release of EVs from astrocytes *in vitro* and this release can be inhibited by antioxidants, like N-Acetyl-L-cysteine or glutaminase inhibitors, highlighting the role of glutaminase in mediating EV release in inflammatory conditions (Wang et al., 2017). Moreover, both IL-1β and TNF-α increase the release of EVs from astrocytes and induce a change in the miRNA load, as reported by Chaudhuri et al. The majority of the miRNAs enriched in the EVs from astrocytes treated with IL-1β and TNF-α target mRNAs supporting neuronal functions, like neurogenesis and synaptogenesis (Chaudhuri et al., 2018). In particular, miR-125a-5p and miR-16-5p target the transmembrane tyrosine kinase receptor for neurotrophin 3, described to promote neuronal survival and differentiation (Bartkowska et al., 2007), and Bcl2 in neurons. These data suggest that inflammatory cytokines induce the release of EVs enriched with miRNAs that reduce dendritic growth in developing neurons, simplify dendritic complexity in mature neurons, and reduce neuronal excitability (Figure 1B), a fact that the authors hypothesized to be protective in conditions of brain inflammation (Chaudhuri et al., 2018). Moreover, IL-1β can alter the proteomic cargo of the astrocyte-derived EVs, leading to an enrichment of proteins, like C3, a component of the complement system, prothymosin alpha and lysyl oxidase, involved in the stimulation of the peripheral immune response (Samara et al., 2017; Datta Chaudhuri et al., 2020).

Some studies propose the effects of astrocyte-derived EVs on neurons in inflammatory conditions as detrimental. A recent work characterized the proteomic profiles of EVs from both non-treated and IL-1β stimulated human astrocytes and analyzed their functional impact on primary mouse cortical neurons (You et al., 2020). In agreement with previous studies, the release of EVs was increased in IL-1β-treated astrocytes with altered proteomic profile. In particular, the authors identified proteins related to pathways characteristic of reactive astrocytes, including cell metabolism and organization, cellular communication and inflammatory response, and a cluster of surface proteins which regulate endocytic pathways. Moreover, they showed that the cellular uptake of EVs by neurons is increased for EVs from reactive astrocytes, that in turn have detrimental effects on neurite differentiation and neuronal firing, contrary to the EVs from the non-treated astrocytes, that promote neuronal maturation (You et al., 2020). In 2015, Mao et al. described that *in vitro* lipopolysaccaride (LPS)-stimulated astrocytes release EVs that increase neuronal apoptosis after treatment with low concentrations of neurotoxins (Mao et al., 2015). These EVs can enter the targeted neurons and carry different miRNAs, compared to EVs isolated from resting astrocytes. EVs from LPS-stimulated astrocytes show the upregulation of miRNAs targeting proteins involved in the regulation of the apoptosis, including the miR-34a, that is able to bind Bcl2 in the neuronal targeted cells, finally reducing its anti-apoptotic functions (Mao et al., 2015).

However, assessing the impact of astrocytes on the inflammatory conditions of neurodegenerative diseases is not easy, as regional diversity may exist, as suggested by a recent work on amyotrophic lateral sclerosis (ALS) (Gomes et al., 2020). ALS is a neurodegenerative disorder in which astrocytes are important players for motor neuron loss in both brain cortex and spinal cord. The authors showed that the inflammation-related miRNAs miR-155, miR-21 and miR-146a are downregulated in primary cortical astrocytes from mSOD1 mice but upregulated in spinal cord astrocytes (Gomes et al., 2020). Interestingly, a reduction of these miRNAs was reported in the EVs from both cortical and spinal cord astrocytes, suggesting the need to assess the impact of astrocyte-derived EVs in ALS-related neuroinflammatory processes.

Binge ethanol drinking in adolescence causes neuroinflammation and brain damage. Interestingly, it has been shown that *in vitro* the treatment of astrocytes with ethanol augment the secretion and alter the content of the EVs, by increasing the cargo of inflammatory proteins involved in the innate immune defense, in particular associated with the TLR4 and NLRP3 pathways, as well as inflammation-related miRNAs (Ibáñez et al., 2019). Astrocyte-derived EVs can be internalized by cortical neurons and affect the physiological state of neurons by altering the expression of inflammation-related proteins and miRNAs. These results support the role of astroglia-derived EVs in promoting neuroinflammation and pinpoint the role of TLR4 in this type of response. Indeed, no ethanol effects were observed on the EVs that derived from the TLR4-KO astrocytes (Ibáñez et al., 2019).

The release of EVs from pro-inflammatory astrocytes was also studied in the presence of systemic inflammation, since systemic immune activation can have an impact on CNS functionality. A 2018 study showed that, following systemic immune activation, mice have increased inflammatory marker levels in the brain and an augmented expression of MHC class I molecules in neurons and astrocytes (Sobue et al., 2018). The authors described that transgenic mice expressing the soluble form of MHCI/H-2D in astrocytes of the medial prefrontal cortex show behavioral alterations, activated microglial cells, decreased parvalbumin-positive cell numbers, and reduced dendritic spine density. The repetitive treatment with GW4869, an agent that inhibits EVs secretion, provided almost a complete protection against behavioral and neuropathological deficits suggesting the possible involvement of EVs in the onset of social and cognitive deficits following immune system activation (Sobue et al., 2018).

### Astrocyte-Derived EVs, Protein Spreading, and Neurotoxic Action in Neurodegenerative Disorders

In neurodegenerative disorders the spreading of pathologic proteins has a central role and EVs have been hypothesized to take part in this process. Astrocytes activate secretory pathways that can selectively eliminate mutant SOD1 and possibly other misfolded or oxidized proteins, to reduce the formation of intracellular toxic aggregates. Indeed, mutant SOD1 primary astrocyte cultures can secrete mutant SOD1-containing EVs. These EVs transfer mutant SOD1 into spinal neuron cultures and selectively induce motor neuron death (Basso et al., 2013). Also studies on animal models support the role of astrocyte-derived EVs in the spread of pathogenic SOD1. For example, Silvermann et al. analyzed the proteome of EVs from neuronal tissue of SOD1G93A mice at the onset of motor neuron disease. They found out that the proteome of brain-derived EVs was largely unchanged compared to the wild-type (WT) mice, but both brain- and spinal cord-derived EVs carry misfolded and aggregated SOD1, and express astrocyte and neuronal markers, (Silverman et al., 2019). The authors showed that also EVs from human SOD1-familial ALS neural tissues carry misfolded and aggregated SOD1, indicating that mutant/misfolded SOD1 within EVs may be a potential mechanism for the systematic spread of the pathogenic protein in ALS. Moreover, probably other factors beside mutant SOD1, can contribute to this toxicity. To this regard, a recent work using induced pluripotent stem cells derived from patients suffering of ALS, carrying the C9orf72 mutation, showed that the astrocytes derived from patients secrete less EVs, compared to healthy controls (Varcianna et al., 2019). Moreover, EVs from the patients' astrocytes are sufficient to induce motoneuron death and the miRNAs typically found to be altered in patients' EVs target transcripts involved in the regulation of axonal/neurite growth and maintenance, thus underlining the detrimental effect of astrocyte-derived EVs in ALS.

The role of EVs has also been analyzed for the spreading of Aβ oligomers and their ability to induce neuronal cell death and impairment of synaptic functions. Söllvander et al. described that, *in vitro*, Aβ_42_ protofibrils are indirectly neurotoxic (Söllvander et al., 2016). Indeed, they can be engulfed by astrocytes, but their degradation is extremely slow and results in long-term intracellular deposits of Aβ. In turn, this accumulation leads to severe lysosomal dysfunctions (Söllvander et al., 2016). Moreover, the accumulated Aβ in the astrocytes can be partially modified to N-terminally truncated Aβ, that is more resistant to degradation, more prone to aggregate and more toxic, compared to the full-length Aβ (De Kimpe et al., 2013). These exposed astrocytes secrete EVs, primarily containing the N-terminally truncated form of Aβ_42_, and they can induce the apoptosis of cultured neurons, thus demonstrating a detrimental role of astrocyte-derived EVs in Alzheimer's disease (AD) (Söllvander et al., 2016).

Pathogenic proteins can have an impact on the release of EVs from astrocytes. For example, EV release is significantly reduced when cultured astrocytes are treated with Aβ_1−42_, probably due to the stimulation of JNK phosphorylation (Abdullah et al., 2016). The reduction in EV release induced by Aβ may increase Aβ accumulation and toxicity, finally leading to an exacerbation of AD pathology (Abdullah et al., 2016). The inhibition of EV release from astrocytes has also been observed for mutant Huntingtin (mHtt), as reduction of EVs released from astrocytes from the HD140Q knock-in mouse model, compared to WT astrocytes, was observed (Hong et al., 2017). In the same work, the authors reported that the misfolded protein are not detected in the EVs isolated from cultured astrocytes and that mHtt can inhibit the expression of αB-crystallin, a protein described mediating exosome secretion, thus reducing the release of EVs.

As mention before, EVs from astrocytes can carry not only misfolded pathogenic protein, but also neurotoxic factors, as it has been described for neurotoxic HIV-related proteins. Indeed, HIV-1 has an impact also in the CNS, inducing neuroinflammation and leading to HIV-associated neurological disorders (Katuri et al., 2019). Several viral proteins can damage the CNS and, among these proteins, Nef has a role in mediating neuronal toxicity. Nef can be expressed in astrocytes and recently it has been described to be delivered to neurons via astrocyte-derived EVs (Sami Saribas et al., 2017). In addition, the transactivator of transcription has a neurotoxic effect and the exposure of astrocytes to this protein leads to an increased expression and release of several miRNAs, through EVs. These EVs can be taken up by hippocampal neurons and lead to the loss of both excitatory and inhibitory synapses (Hu et al., 2019).

## Role of Oligodendrocyte-Derived EVs in the Central Nervous System

Myelin is the membrane wrapping the axons of neurons in the CNS, allowing the saltatory conduction which is vital for the correct transmission of the nerve impulse. Myelin homeostasis is related to brain plasticity and learning, and its dysregulation is associated to neuro-psychiatric disorders and age-related cognitive decline (McKenzie et al., 2014; Hasan et al., 2019). Oligodendrocytes are highly specialized cells responsible for myelin production; they derive from the oligodendrocyte precursor cells (OPCs) which can maintain the ability to proliferate and differentiate in myelinating oligodendrocytes in the adult CNS, allowing myelin homeostasis and physiological regeneration (Kang et al., 2010). Oligodendrocytes are affected by neuronal activity, that influence different aspects of oligodendrocyte lineage progression, like proliferation, differentiation, survival and myelination (Barres and Raff, 1993; Almeida and Lyons, 2016). Moreover, oligodendrocyte function goes beyond the mere production of myelin in fact they contribute, together with the other glial cells, to the regulation of network formation, and to the maintenance of its correct functioning (Allen and Lyons, 2018; Suminaite et al., 2019). In addition, oligodendrocytes provide axons with external energy substrates, such as lactate (Fünfschilling et al., 2012; Lee et al., 2012).

### Effects of Oligodendrocyte-Derived EVs on Neurons

As for the astrocytes, also oligodendrocytes secrete EVs. A 2007 work in primary cultured oligodendrocytes, in absence of neurons, showed that EV secretion from oligodendrocytes is Ca^2+^-dependent and the released EVs carry unprocessed proteolipid protein (PLP) and DM20, two abundant proteins of myelin which are produced by alternative splicing from the PLP gene (Krämer-Albers et al., 2007). Based on this observation, the authors suggested that the function of oligodendroglial EVs might be the disposal of redundant myelin components produced by default by oligodendrocytes to balance myelin production *in vivo*. Moreover, EVs from oligodendrocytes not only contain myelin proteins, but they carry a plethora of chaperones and enzymes involved in the management of oxidative stress, raising the intriguing suggestion that also oligodendroglial EVs have a role in transcellular signaling between glia cells and neurons (Krämer-Albers et al., 2007).

**Figure 2:**
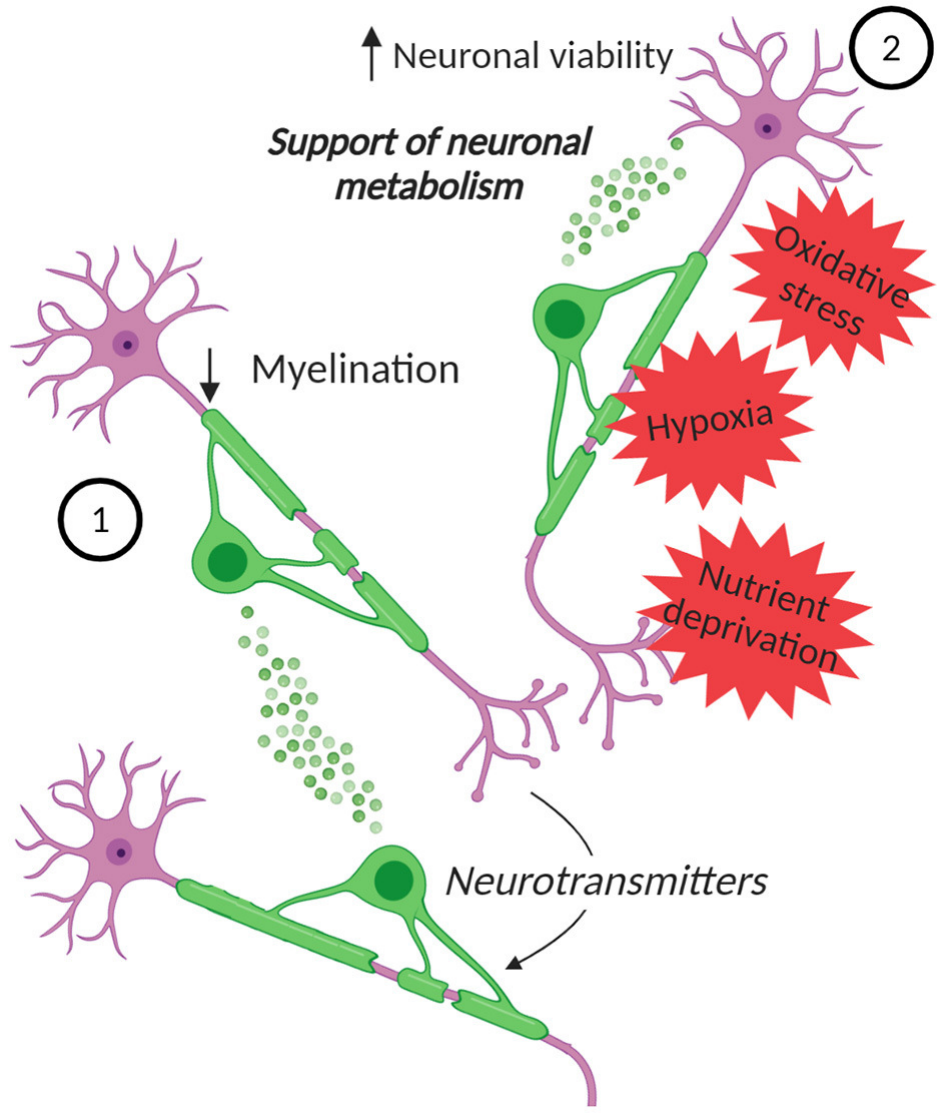
EVs in oligodendrocyte-neuron communication. Neurons can control the secretion of EVs from oligodendrocytes. (1) These EVs can inhibit myelination, by reducing oligodendrocyte surface expansion. (2) Moreover, the EVs can influence neurons, supporting their metabolism and increasing their viability under stress conditions, such as oxidative stress, starvation, and OGD. Created with BioRender.com.

The release of EVs from oligodendrocytes, that is regulated by neurotransmitters, like glutamate, released from the neurons, and the role of these EVs in glia-neurons signaling was confirmed by later studies. An *in vitro* work supported the fact that neurons control the secretion of EVs from oligodendrocytes and showed that EVs themselves can inhibit myelination (Bakhti et al., 2011) (Figure 2). This is possible since EVs can have an autoinhibitory effect by reducing oligodendrocyte surface expansion. An additional study presented a model in which activity-triggered EV release from myelinating oligodendrocytes occurs along internodes and paranodal regions into the periaxonal space, where they are internalized by axons. Furthermore, EV release may also occur from cell bodies and their uptake can also take place at the level of neuronal somas or dendrites (Frühbeis et al., 2013). Importantly, this work presented not only *in vitro*, but also *in vivo* evidence for the uptake of oligodendrocyte-derived EVs by neurons and showed the functional recovery of their cargo. Furthermore, the supply of cultured neurons with oligodendroglial EVs supports the neuronal metabolism and increases neuronal viability under conditions of cell stress. Indeed, neurons treated with oligodendroglial EVs were less sensitive to oxidative stress or starvation (Frühbeis et al., 2013). In addition, the treatment with oligodendrocyte-derived EVs protects neurons during OGD (Fröhlich et al., 2014) (Figure 2). Although a complete characterization of the cargo molecules involved in these protective mechanisms is still lacking, the presence of Hsc/Hsp70, SOD1 and catalase has been described and these molecules can be transferred to neurons (Frühbeis et al., 2013; Fröhlich et al., 2014). These proteins are involved in the antioxidant defense and have a neuroprotective effect. Furthermore, in the targeted neurons several kinases are activated upon treatment with oligodendroglial EVs, including Akt, Erk1/2, and JNK, proteins that are implicated in promoting cell survival and in several physiological processes, including neuronal functions and cell death/survival pathways (Dudek et al., 1997; Jin et al., 2002; Zeke et al., 2016). Also the transcription factor CREB and the enzymes GSK-3α/β and GSK-3β are phosphorylated, highlighting the variety of pathways potentially involved in mediating the neuroprotective effect of oligodendrocyte-derived EVs on neurons (Fröhlich et al., 2014). Moreover, *in vitro*, oligodendroglial EVs enhance the spontaneous neuronal activity of treated neurons, but the impact of EVs on neuronal excitability *in vivo* remains to be proven.

## Role of Microglia-Derived EVs in the Central Nervous System

Microglia constitute the resident immune cells of the CNS, constantly monitoring and responding to changes in the local environment. Microglia have been traditionally divided into the pro-inflammatory M1 and the anti-inflammatory M2 microglia, yet any classification over simplify the real complexity of these cells (Ransohoff, 2016). Microglia respond to tissue injury or to the presence of pathogens and switch from a “resting” homeostatic state to an active state. Microglia have several other essential functions and they sense neuronal activity mediated by the expression of membrane receptors for several neurotransmitter, whose activation influences key microglial functions (Marinelli et al., 2019). In turn, they can release molecules that can bind to receptors on neurons, contributing to the control of neurotransmission and thus allowing the bidirectional neuronal-microglial communication essential for sculpting of neuronal connections during development and, later, for the maintenance of CNS homeostasis (Marinelli et al., 2019). In the adult CNS, the chemokine signaling between neurons and microglia, and the appropriate levels of cytokines are essential for maintenance of normal brain plasticity (Stellwagen and Malenka, 2006; Rogers et al., 2011). Furthermore, it is known that inflammatory *stimuli* can alter synaptic plasticity, leading to aberrant synaptic depression or potentiation, based on the type of the *stimulus* (Costello et al., 2011; Pascual et al., 2012; Zhang et al., 2014). In this way, microglia and their secreted cytokines can have a direct impact on CNS development, learning, and memory (Depino et al., 2004; Yli-Karjanmaa et al., 2019).

### Effects of Microglia-Derived EVs on Neurons

**Figure 3:**
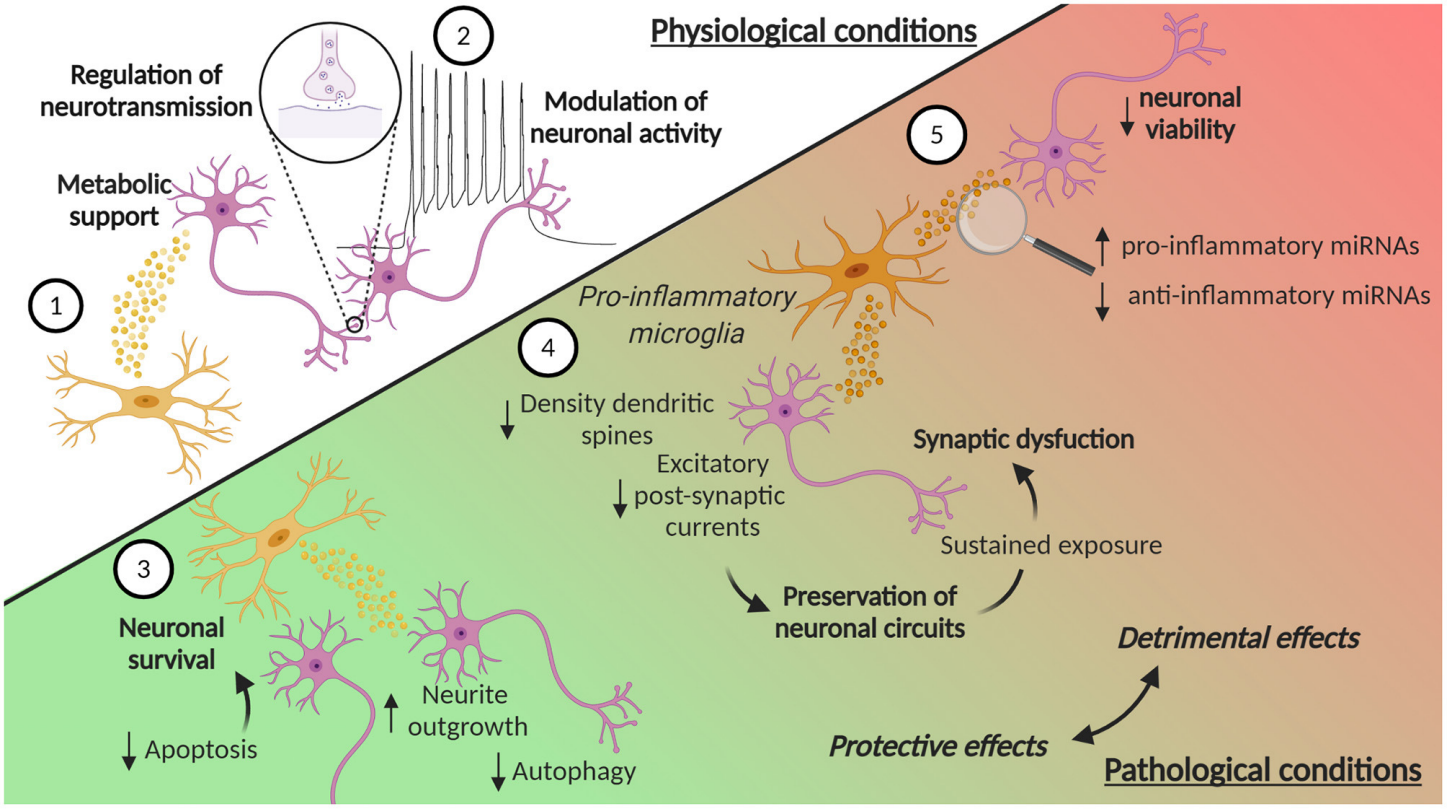
EVs in microglia-neuron communication in physiological and pathological conditions. (1) Microglia can release EVs that contribute to the metabolic support of neurons. Moreover, (2) EVs from microglia can contribute to the homeostatic regulation of neurotransmission and modulate neuronal activity, by enhancing spontaneous and evoked excitatory transmission. Microglia-derived EVs are also involved in pathological conditions, where they can play a dual role, protective or detrimental. In ischemic stroke, (3) microglia-derived EVs can reduce neuronal apoptosis, thus increasing neuronal survival, while in TBI they promote neurite outgrowth and inhibits autophagic activity. (4) EVs from microglia treated with pro-inflammatory *stimuli* reduce the density of dendritic spines and decrease excitatory postsynaptic currents. These synaptic changes may represent a compensatory response to preserve neuronal circuits and counteract hyperexcitability induced by pro-inflammatory cytokines. However, the sustained exposure of neurons to these EVs leads to an over-inhibition of synaptic function and to an excessive synapse destabilization, contributing to synaptic dysfunction typical of chronic neuroinflammation. (5) EVs from pro-inflammatory microglia can also contribute to neuroinflammation, carrying increased levels of pro-inflammatory miRNAs and reduced levels of anti-inflammatory miRNAs. Moreover, EVs from pro-inflammatory microglia induce a reduction in neuronal viability. Created with BioRender.com.

Not only chemokines and cytokines are important for the microglia-neuron communication, but an increasing number of evidence supports the key role of EVs. Microglia secrete EVs that contribute to the metabolic support of neurons: they contain enzymes essential for anaerobic glycolysis and lactate production that could contribute as supplementary energy substrate during synaptic activity (Potolicchio et al., 2005) (Figure 3). Moreover, the presence of enzymes involved in the glycolysis, like pyruvate kinase and glyceraldehyde 3 phosphate dehydrogenase, has also been reported, together with members of the heat shock protein family, molecular chaperones important for cell survival (Potolicchio et al., 2005; Hooper et al., 2012). In addition, EVs from cultivated microglia contain enzymes involved in protein degradation, suggesting that microglial EVs contribute to the catabolism of neuropeptides (Potolicchio et al., 2005). Moreover, microglia can be stimulated to produce EVs by different signals. For example, Hooper et al. described that primary rat microglia release EVs following stimulation with Wnt3a, but not under control conditions (Hooper et al., 2012). Furthermore, serotonin secreted from neurons can bind to microglial 5-HT receptors and induce the elevation of intracellular Ca^2+^ levels, thus finally stimulating EV release (Glebov et al., 2015).

Microglia-derived EVs have also an impact on neuronal functionality in the context of neurological disorders. To this regard, a 2019 study showed that EVs from BV2 cells treated with IL-4 are neuroprotective in ischemic stroke, augmenting neuronal survival, and through other factors, such as fibroblast growth factor, nerve growth factor, and brain-derived neurotrophic factor (BDNF) (Song et al., 2019) (Figure 3). EVs from microglia treated with IL-4 are taken up by neurons both *in vitro* and *in vivo*, and contribute to neuronal survival due to the transport of elevated levels of miR-124. This miRNA is linked to hypoxic injury and its expression can attenuate neuronal damage by reducing M1 macrophage activation and promoting the anti-inflammatory microglia phenotype (Ponomarev et al., 2011; Veremeyko et al., 2013). The transfer of miR-124 is neuroprotective, probably by targeting the expression of ROCK and USP14 and thereby reducing neuronal apoptosis in mice subjected to tMCAO (Song et al., 2019). Interestingly, the increase in miR-124 levels has also been described in TBI (Huang et al., 2018; Li et al., 2019). miR-124-3p levels in microglia-derived EVs increase during the acute to the chronic phase transition after TBI. The transfer of miR-124-3p from EVs promotes also neurite outgrowth in scratch-injured neurons (Huang et al., 2018) and inhibits autophagic activity (Li et al., 2019) (Figure 3).

#### Impact of Microglia-Derived EVs on Neuronal Morphology and Synaptic Plasticity

Microglia can modulate neuronal activity via release of EVs: EVs from cultured primary rat microglia and N9 cell line can interact with the plasma membrane of cultured hippocampal neurons, thus enhancing spontaneous and evoked excitatory transmission (Antonucci et al., 2012) (Figure 3). Although this work lacks an extensive characterization of EVs, it suggested that EVs may influence neurotransmission by inducing the metabolism of sphingolipids, molecules described to be involved in the release of neurotransmitters (Mochel, 2018). Indeed, EV treatment leads to the increase of the formation of sphingosine and ceramide from sphingomyelin (Antonucci et al., 2012).

The impact of microglia-derived EVs on synaptic transmission has also been described in pathologic conditions, like neuropathic pain. Following the spinal nerve ligation, the release of EVs from microglia augment in a time-dependent manner and these EVs promote the release of the pro-inflammatory IL-1β and enhance the frequency and the amplitude of excitatory postsynaptic currents (Li et al., 2017). The impact of microglial EVs on neuronal activity has also been described in the context of metabolic dysfunctions: primary rat microglia incubated with the saturated free fatty acid palmitate not only increase the release of cytokines, but their released EVs can induce morphologic alterations to dendritic spines in hippocampal cultured neurons (Vinuesa et al., 2019). These dendritic spines are more immature and thinner, suggesting the involvement of microglial EVs in neuronal dysfunction upon metabolic insult.

The effect of microglia EVs on synapses may be mediated again by specific miRNAs. For example, miR-146a-5p is upregulated in the EVs isolated from reactive primary rat microglia, treated with inflammatory and degenerative *stimuli*, and can be transferred to neurons (Prada et al., 2018). It is known that miR-146a-5p represses the translation of the presynaptic Synaptotagmin-1 and the postsynaptic Neuroligin-1 proteins (Jovičić et al., 2013), and, in accordance, the study by Prada et al. showed a reduction of both of these proteins in neurons treated with microglia-derived EVs containing miR-146-5p (Prada et al., 2018). Moreover, the neurons showed a reduction in the density of dendritic spines and a decrease in excitatory postsynaptic currents. These synaptic changes may represent a compensatory response to preserve neuronal circuit, and counteract acute hyperexcitability and excessive glutamatergic transmission, induced by pro-inflammatory cytokines released by microglia (Figure 3). However, sustained exposure of neurons to EVs containing miR-146a-5p leads to an over-inhibition of synaptic function and to an excessive synapse destabilization, leading to the pathological loss of synapses, a situation that may contribute to synaptic dysfunction typical of chronic neuroinflammation (Prada et al., 2018) (Figure 3). Interestingly, miR-146a-5p is present, together with other miRNAs, in EVs isolated from the CSF of patients suffering of multiple sclerosis (MS), proving an evidence for the possible link between microglia activation, enhanced EV production and cognitive symptoms in MS patients (Prada et al., 2018).

MiRNAs are not the only molecules carried by the EVs that can mediate an effect on synapses. The N-arachidonoylethanolamine, an endocannabinoid, has been described to be present and enriched on the surface of EVs released from microglia (Gabrielli et al., 2015). EVs carrying this endocannabinoid can activate the type-1 cannabinoid receptor in primary hippocampal cultures, inhibiting GABA release and miniature inhibitory postsynaptic currents, thus contributing to the homeostatic regulation of neurotransmission (Gabrielli et al., 2015).

### Effects of Pro-inflammatory Microglia Derived EVs on CNS Cells

Microglia in different conditions can secrete distinct EV populations. EVs from pro-inflammatory microglia have been described to contribute to neuroinflammation. The overexpression of glutaminase C (GAC), present in early stages of AD-like pathology in mouse brains, promotes microglial pro-inflammatory activation and induces release of EVs that contain increased levels of pro-inflammatory miRNAs and decreased levels of anti-inflammatory miRNAs (Gao et al., 2019) (Figure 3). Activated microglial BV2 cells, stimulated with LPS, release more and larger EVs, that contain higher levels of pro-inflammatory cytokines, like IL-6 and TNF, and have a distinct proteomic profile (Yang et al., 2018). Moreover, it has been shown that EVs released from LPS-treated microglia induce a reduction in cell viability in cultured neurons, suggesting a detrimental effect of these vesicles (Tang et al., 2016) (Figure 3). In this study, the authors highlighted that LPS-treated microglia show an augmented miR-375 expression, an increase also described at the level of their EVs. miR-375, a miRNA involved in cell viability and apoptosis, may reduce Pdk1 protein expression in cultured neurons, suggesting its involvement in the EV-induced neuronal damage. Another work, focused on clarifying the role of the metabotropic glutamate receptor 5 (mGlu5) in microglia activation, support the detrimental role of microglia-secreted EVs (Beneventano et al., 2017). In BV2 cells, the current elicited by the P2X7 receptor activation is increased following stimulation of the mGlu5 receptor with an agonist and this treatment leads to an augmented shedding of EVs. The authors reported that the stimulation of mGlu5 receptor increase the expression of miR-146a (Beneventano et al., 2017), a miRNA up-regulated by pro-inflammatory signals and that negatively controls a plethora of genes involved in inflammation (Lukiw et al., 2008; Li et al., 2011). Moreover, upon P2X7 receptor stimulation, this miRNA can be packed into the released EVs, thus reaching the neighboring cells. In addition, the stimulation of microglia with LPS leads to increased miR-146a levels in the EVs (Beneventano et al., 2017).

On the other hand, in the context of CNS tumor, EVs from microglia treated with LPS and IFN-γ may have a protective effect. In fact, these EVs can interfere with the migration and invasion of glioma cells *in vitro*. When injected into the brain of mice with glioma, these EVs led to a decrease in the tumor size by reducing the proliferation and migration of cells in the tumoral region (Grimaldi et al., 2019). In addition, the EVs can act on the tumor-associated myeloid cells and direct them toward an antitumor phenotype, highlighting the ability of microglia to communicate with several cell types through the release of EVs. This underlines how the impact of EVs from microglia subjected to a pro-inflammatory environment can be detrimental or beneficial depending on the context considered, supporting the need to study the involvement of microglial EVs in different pathological conditions in more detail.

### Microglia-Derived EVs and Protein Spreading in Neurodegenerative Disorders

The involvement of microglia-derived EVs in the pathogenesis of neurodegenerative disorders appears to go beyond their role in neuroinflammation, and they are also implicated in the spatiotemporal propagation of pathogenic proteins. In AD, EVs can act as an endogenous source of lipids able to shift the equilibrium toward toxic Aβ species. Indeed, EVs released from primary rat microglia increase Aβ toxicity in cultured hippocampal neurons: the lipid components of the EVs promote the formation of small soluble neurotoxic species from Aβ_1−42_ extracellular aggregates, that can activate *in vitro* the NMDA receptor activity, finally inducing excitotoxic damage (Joshi et al., 2014). In addition, microglial EVs contain toxic forms of Aβ_1−42_ and Aβ_1−40_, supporting a detrimental role of microglia in AD that contribute to the spreading of neurotoxic amyloid species throughout the brain. It has been hypothesized that EV-mediated release of neurotoxic Aβ species may occur when the intracellular pathways of Aβ degradation are saturated, in this way, the shedding of EVs may become a way for microglia to eliminate Aβ (Joshi et al., 2014). The observation that microglia-derived EVs collected from the CSF of AD patients promote the extracellular formation of neurotoxic Aβ species *in vitro* further supports the involvement of EVs in Aβ spreading. However, additional studies will be essential to define the topology of Aβ species and to clarify whether Aβ forms are associated to the extracellular membrane of shed EVs. Moreover, it is important to underscore that EVs involved in Aβ spreading are also shed by neurons and they may accelerate Aβ amyloidogenesis *in vitro* (Yuyama et al., 2012). These EVs can be taken up by microglia for degradation, highlighting how microglia can have a double positive and detrimental role in AD, also in this context, and the importance of the constant coordination between neurons and microglia.

Tau is present in EVs isolated from the PS19 mouse brain, a mouse model of tauopathy (Asai et al., 2015). Microglia depletion reduces the content of tau in brain EVs and suppresses the transmission of tau containing EVs to neurons *ex vivo*, suggesting that microglia-secreted EVs spread tau. However, it is important to note that the transmission of tau *via* microglia-derived EVs is not the only mechanism involved in the detrimental protein spreading. Asai et al. suggested that the entorhinal cortex, one of the first regions affected by tau accumulation, is less dependent on EV-mediated tau propagation, whereas this mechanism seems to be more involved in tau accumulation in the dentate gyrus (Asai et al., 2015).

Microglial EVs take part not only to the spreading of pathologic proteins in AD, but they may also be involved in α-synuclein (α-syn) transmission in PD. EVs isolated from the plasma of PD patients show higher total α-syn and α-syn oligomers levels, compared to healthy controls (Xia et al., 2019). Injection of these EVs into the striatum of mice reveal that EVs can spread throughout the brain and they can be taken up by microglia. In turn, microglia can secrete α-syn via EVs and the pathogenic protein can be transmitted to neurons, thus contributing to its spreading into the brain.

All these observations suggest an important role of EVs in the transmission of pathogenic proteins, thus placing EVs as central contributors to the pathogenesis of neurodegenerative disorders. However, further studies to clearly understand EVs role in protein spreading, but also to shed light on how these proteins are internalized by the recipient cells, will be essential to guide future research to develop effective treatments able to block the pathological protein spreading.

## Extracellular Vesicles in Glia-to-Glia Communication

The EVs secreted from glial cells do not only have an impact on neurons but can target other glial cells. It has been reported that microglia uptake EVs released from astrocytes treated with morphine, leading to impaired phagocytosis (Hu et al., 2018). Moreover, a recent study on TBI revealed that astrocytes can regulate microglia polarization by releasing EVs (Long et al., 2020). In particular, the EVs released from astrocytes exposed to TBI brain extract are enriched with miR-873a-5p and they can promote microglia anti-inflammatory phenotype transformation, suppressing pro-inflammatory factors and promoting the release of anti-inflammatory molecules, due to the inhibition of ERK and NF-κB, thus finally contributing to the attenuation of brain edema (Long et al., 2020). In addition, a study on cellular aging highlighted how astrocyte-derived EVs can target oligodendrocytes (Willis et al., 2020). The authors described astrocyte-derived EVs as key contributors to the cellular effects of the inflammatory phenotype of aged cells: EVs from young astrocytes, but not from aged astrocytes, can transfer oligodendrocyte progenitor cells proteins which contribute to their maturation to oligodendrocytes (Willis et al., 2020).

Moreover, it is well-known that microglia strongly influence remyelination, with a predominant pro-regenerative phenotype essential for an efficient myelin repair after damage (Miron et al., 2013). However, how microglia can have an impact on myelin repair was unclear. A recent study described that EVs from pro-inflammatory microglia can block remyelination, contrary to the EVs released by pro-regenerative microglia, that are able to promote OPC recruitment and differentiation (Lombardi et al., 2019). Interestingly, this study highlighted the fact that astrocytes mediate the detrimental action of inflammatory microglia-derived EVs on OPCs. *In vitro*, the authors described how EVs from pro-inflammatory microglia favor differentiation of OPCs, but, when co-cultured with astrocytes, they block OPC maturation (Lombardi et al., 2019). This observation is important in the context of demyelinating disorders, like MS, because it can be hypothesized that demyelinating lesions fail to remyelinate because of the release of EVs from chronically activated microglia, that can block OPC differentiation, by inducing harmful astrocyte conversion, thus nullifying the direct pro-myelinating action of EVs (Lombardi et al., 2019).

## Possible Therapeutic Strategies for CNS Disorders Based on Extracellular Vesicles

Development of EV based therapeutics for neurological conditions, in which the crossing of the BBB has always represented an obstacle to overcome, has been recently under active investigation. Poor brain penetration of many drugs ranging from small molecules to bioactive proteins constitute a problem. Interestingly, Yuan et al. showed improved targeting of BDNF to the brain parenchyma when complexed with EVs, compared to free BDNF, highlighting the feasibility of using EVs as drug delivery vehicles (Yuan et al., 2017). EV tissue distribution has been shown to be dependent on the cellular origin of the EVs (Wiklander et al., 2015). While dendritic cell (DC)-derived EVs distributed more to the spleen, melanoma- or muscle cell-derived EVs were found in higher levels in the liver. Alvarez-Erviti et al. were the first to demonstrate improved brain delivery of EVs from DCs expressing the rabies viral glycoprotein (RVG), a protein that specifically binds to the acetylcholine receptor, on EV surface (Alvarez-Erviti et al., 2011). In this and in a later study (Wiklander et al., 2015), EVs from DCs carrying RVG were intravenously administered to C57BL/6 mice and efficiently reached the brain. This technological demonstration serves as an intriguing platform to further improve the targeting and bioavailability of EVs to possible specifically delivery drugs to the CNS. EVs can further be loaded with therapeutic proteins (Haney et al., 2015; Patel et al., 2018). Moreover, Kojima et al. designed a method to boost EV production in a desired cell type and to increase the loading of specific mRNAs. The brain delivery by this system was ensured by RVG on EV surface. The engineered cells were implanted subcutaneously in mice and the delivery of the catalase mRNA by the designed EVs attenuated neurotoxicity and neuroinflammation, indicating the potential usefulness of the method as a therapeutic application (Kojima et al., 2018).

Interestingly, neuroinflammation, induced by intracranial LPS injection, increases the distribution of EVs in the brain (Yuan et al., 2017). This is an important factor to consider in several neurogenerative diseases with prominent neuroinflammation as part of the pathology, suggesting that EVs may have a good bioavailability in the brain of the patients. Indeed, intranasally administered EVs increasingly accumulate in the brain of mice after focal brain ischemia, compared to the healthy brains (Betzer et al., 2017). In this study, the authors demonstrated a more efficient brain delivery of EVs via intranasal compared to intravenous administration, highlighting the possibility for less invasive administration routes. Similar enhancement of brain delivery of intranasally administered EVs is observed in the context of kainic acid induced seizures (Kodali et al., 2020), where human mesenchymal stem cell (MSC)-derived EVs distribute throughout the brain and co-localize with neurons and microglia. In addition, the authors proposed that the uptake of the EVs may be higher in injured cells compared to the healthy ones, providing an extra level of pathology specific targeting. However, the BBB of mice and humans have been shown to react differently in response to drug treatments (Jackson et al., 2017), implicating that crucial species differences in the function of the BBB, and targeting of EVs to the brain, may exist. Brain distribution studies of EVs in humans or primates are still lacking.

Whilst mainstream of studies have focused on using MSC-derived EVs as therapeutic vehicles (Xin et al., 2017; Losurdo et al., 2020), the use of EVs from glial cells to treat neurological disorders has been also tested. Astrocyte-derived EVs injected intravenously in a model of MCAO enhance neuronal viability and inhibit the activation of astrocytes and microglia (Pei et al., 2019). The beneficial effects of astrocyte-derived EVs have been described also in a rat model of TBI (Chen et al., 2020). Injection of EVs from WT astrocytes into the striatum of a mouse model of HD reduces the density of mHtt aggregates in the injected area, thus highlighting a role of astrocyte-derived EV in preventing the accumulation of mHtt (Hong et al., 2017). Immediately after MCAO, intravenously injected EVs from IL-4 treated microglia reduced the infarct volume by decreasing neuronal apoptosis, and attenuated behavioral deficits 3 days after the ischemia, probably due to the neuroprotective role of miR-124 (Song et al., 2019). The beneficial effects of this microRNA have been reported also by other studies on TBI: mice with repetitive TBI injected with microglia-derived EVs have a better neurologic outcome and a reduction of neuroinflammation (Huang et al., 2018; Li et al., 2019).

Taken together, these studies suggest that glia-derived EVs may have therapeutic potential. Yet, further studies are essential in order to understand the role of glial EVs in different pathological conditions. It is important to note that in some neurodegenerative diseases, such as AD and PD, glia-derived EVs may be detrimental in contributing to the spreading of misfolded proteins.

In the last years, several studies have focused on the use of EVs as biomarkers to obtain information about the presence and/or the molecular evolution of neoplastic diseases (Le Rhun et al., 2020; Murgoci et al., 2020a). In addition, the use of EVs for glioma therapy has been explored. Murgoci et al. described a method for the isolation and characterization of microglia-derived EVs from rat neonatal cortex, that were used to treat a 3D glioma model (Murgoci et al., 2018). The authors described the ability of these EVs to suppress the invasive behavior of cancer cells, suggesting the use of glia-derived EVs as potential therapeutic agents against glioma. However, in the complex context of tumoral environment, it is essential to consider that several cells can release EVs and have an impact on the tumor. For example, normal human astrocytes, when stimulated by glioma cells, increase the EV levels of MGMT mRNA, encoding the O6-alkylguanine DNA alkyltransferase. The delivery of this mRNA to MGMT-negative glioma cells may confer the resistance to temozolomide, through the repression of apoptosis (Yu et al., 2018). Thus, blocking the mutual exchange of EVs from astrocytes to glioma cells may be a novel strategy to prevent tumor recurrence.

Further studies are needed to deepen the knowledge on the roles of glia-derived EVs in neurodegenerative disorders and in CNS tumors, and to understand if they may potentially constitute an advantage when designing novel therapeutics.

## Conclusion and Future Perspectives

In this review we have summarized the current knowledge on the involvement of glial EVs in the communication between CNS cells. These vesicles can transfer specific molecules that are involved in both physiological and pathological processes, having an impact on the function of the surrounding neurons and glial cells. Owing to the recent advances in high-throughput technology, the screening for RNAs and proteomic components of glial EVs have allowed the identification of several functionally relevant EV-associated molecules (Murgoci et al., 2020b). However, despite the recent efforts, we are still far from complete understanding the role of glial EVs in health and disease and future studies are essential to clarify the functional effects of these vesicles and the specific modes of actions. Furthermore, it is worth noting that cell specific EVs can be studied *in vitro*, but there are still methodological difficulties to study the EV biology *in vivo*. Thus, efforts should be put into development of new models and more powerful imaging and tracking methods to allow studies *in vivo*. In addition, special emphasis should still be placed for the development of standardized protocols, useful to promote harmonization among laboratories and to allow a tighter control for EV characterization, an essential requirement for all EV studies.

## Author Contributions

CP wrote the manuscript and prepared the figures. NB contributed to manuscript writing, discussed ideas, and helped in the correction. IS contributed to manuscript writing. TM critically revised the manuscript. All authors contributed to the article and approved the submitted version.

## Conflict of Interest

The authors declare that the research was conducted in the absence of any commercial or financial relationships that could be construed as a potential conflict of interest.
